# Can S100B Predict Cerebral Vasospasms in Patients Suffering from Subarachnoid Hemorrhage?

**DOI:** 10.3389/fneur.2013.00065

**Published:** 2013-06-06

**Authors:** Moshgan Amiri, Ramona Astrand, Bertil Romner

**Affiliations:** ^1^Department of Neurosurgery, Rigshospitalet, University Hospital of Copenhagen, Copenhagen, Denmark; ^2^Faculty of Medicine, Copenhagen University, Copenhagen, Denmark

**Keywords:** protein S100B, subarachnoid hemorrhage, cerebral vasospasm, CT angiography, cerebrospinal fluid, serum

## Abstract

**Background:** Protein S100B has proven to be a useful biomarker for cerebral damages. Increased levels of serum and cerebrospinal fluid (CSF) S100B have been shown in patients suffering subarachnoid hemorrhage (SAH), severe head injury and stroke. In patients with SAH, the course of S100B levels has been correlated with neurological deficits and outcome. Cerebral vasospasm is a major contributor to morbidity and mortality. The primary aim of this study was to investigate the potential of S100B protein as a predictor of cerebral vasospasm in patients with severe SAH.

**Materials and Methods:** Patients with SAH, Fisher grade 3 and 4, were included in the study. Five samples of CSF and serum S100B were collected from each patient. The first sample (baseline sample) was drawn within the first 3 days following ictus and the following four samples, once a day on days 5–8, with day of ictus defined as day 1. Clinical suspicion of cerebral vasospasm confirmed by computed tomography angiography was used to diagnose cerebral vasospasm.

**Results:** A total of 18 patients were included. Five patients (28%) developed cerebral vasospasm, two (11%) developed ventriculitis. There were no significant differences between S100B for those with and without vasospasm. Serum S100B levels in patients with vasospasm were slightly lower within the first 5 days following ictus, compared to patients without vasospasm. Two out of five patients had elevated and increasing serum S100B prior to vasospasm. Only one showed a peak level of S100B 1 day before vasospasm could be diagnosed. Due to the low number of patients in the study, statistical significance could not be reached.

**Conclusion:** Neither serum nor CSF S100B can be used as predictor of cerebral vasospasm in patients suffering from SAH.

## Introduction

Subarachnoid hemorrhage (SAH) accounts for approximately 6–8% of all strokes, and the leading cause is rupture of intracerebral aneurysms in 85% of the cases. The remaining causes are due to arteriovenous malformation (AVM) bleeding, vertebral artery dissection (about 5% together), or due to more undefined causes or the perimesencephalic SAH, which account for about 10% of the cases (van Gijn and Rinkel, 2001). The overall incidence of SAH is approximately 9 per 100,000 persons/year, slightly higher in the Scandinavian countries, an highest in Finland and Japan with 19.7 and 22.7 per 100,000/year, respectively (de Rooij et al., 2007). Mortality and morbidity is high, accounting for up to 50% in patients suffering from aneurysmal SAH (aSAH). About 25% never reach medical attention (Diringer, 2009).

Cerebral vasospasm is an important cause of morbidity and death after SAH (Rowland et al., 2012), for those who survive to receive medical treatment. It is defined as clinical neurological symptoms of ischemia (confusion, decreased level of consciousness, focal neurological deficits), with narrowing of cerebral vessels, visualized by computed tomography angiography (CTA). Cerebral vasospasm occur in approximately one third of aSAH patients (Frontera et al., 2009), and the risk of vasospasm is related to the thickness and amount of blood in the subarachnoid space and/or the presence of intraventricular blood assessed on computed tomography (CT), the Fisher grade (Fisher et al., 1980; Jung et al., 2012). The risk of developing cerebral vasospasm is highest during dag 6–8 following ictus (Weir et al., 1978). SAH patients are clinically monitored with daily neurological examinations, but the means of diagnosing cerebral vasospasm varies. The techniques in use for diagnosing vasospasm are by means of clinical evaluation, transcranial doppler sonography (TCD), CTA, digital subtraction angiography (DSA), or by computed tomography perfusion (CTP) (Yoon et al., 2006; Washington and Zipfel, 2011; Kunze et al., 2012), although the most relevant technique is still not defined (Frontera et al., 2009).

A biomarker for detection of cerebral vasospasm in patients with SAH, could ideally allow for early detection to prevent delayed ischemic neurological deficits. Protein S100B is a calcium binding protein, predominant in nervous tissue, and mainly expressed in astroglial cells (Donato, 2001). It is also found in extracerebral tissues, such as long bones, fat, melanocytes, heart, and kidneys, though in lesser extent (Anderson et al., 2001; Unden et al., 2005). S100B is increased in serum and in cerebrospinal fluid (CSF) after brain injury, mainly as a result of the opening of the blood brain barrier (Marchi et al., 2003). In recent years, studies have shown that S100B is useful as a predictive marker for outcome after cerebral infarction (Herrmann and Ehrenreich, 2003; Ahmad et al., 2012), anoxic brain injury (Shinozaki et al., 2009), and SAH (Wiesmann et al., 1997; Stranjalis et al., 2007; Sanchez-Pena et al., 2008).

The aim of our study was to investigate the potential of S100B protein as a predictor of cerebral vasospasm in patients suffering from severe SAH.

## Materials and Methods

### Patients

We prospectively studied patients with SAH, admitted to Copenhagen University Hospital, neurointensive care unit (NICU) between September 2012 and January 2013. The inclusion criteria were: patients with SAH confirmed by CT, Fisher grade 3 and 4, age 18 years and above, admission and external ventricular drain (EVD) within 3 days of ictus, no other major injuries. Patients who had their EVD removed within the first 8 days after ictus were excluded from the study.

Glasgow Coma Scale (GCS) score (Teasdale and Jennett, 1974) and WFNS grading scale (Teasdale et al., 1988) was used to assess the neurological status on admission. Location and thickness of the hemorrhage on CT scan was determined by the Fisher grading scale (Fisher et al., 1980).

### Samples

A total of five blood samples and five CSF samples were obtained from each patient. The day of ictus was defined as day 1. The first blood and CSF samples (Baseline sample) were obtained between day 1 and 3, depending on when the patient was transferred to the NICU and had received an EVD. The following four samples were obtained on day 5–8 following ictus. Each blood sample consisted of 4 ml of blood obtained in a SST-tube with separating gel without additives, and the CSF samples consisted of minimum 1 ml of CSF obtained from the EVD. About 4 ml of blood and 1 ml of CSF was taken as waste prior to sampling, as a precaution to contamination and dilution effect. Following collection all samples were centrifuged at 3500 rpm for 7 min at room temperature. The separated serum and CSF samples were stored at −80 °C, and thawed prior to analysis. Samples were analyzed with the method of electrochemiluminescence immunoassay, and the equipment used was Elecsys 2010, Modular Analytics E170, Roche Diagnostics (Mannheim, Germany). The lower detection limit was 0.005 and the upper limit 39 μg/L.

### Vasospasm

Daily neurological status was assessed to determine clinical signs of worsening with cerebral vasospasm as the primary cause. Cerebral vasospasm was confirmed by CTA.

### Statistics

Statistical analysis was performed in IBM SPSS Statistics ver. 19.0.0. Figures and tables were computed in Microsoft Excel 2007. Student *t*-test was performed for comparing mean S100B levels between patients with and without cerebral vasospasm. Significance level was set to 0.05.

### Ethical considerations

The study and the collection of S100B in both serum and CSF had been approved by the local ethics committee.

## Results

A total of 18 patients with severe SAH were included, 16 had an aSAH, one had SAH due to a small AVM bleeding, and one due to dissection of the vertebral artery. Mean age was 60 years (range 42–84 years), there were 11 females and 7 males. The mean GCS score on admission was 8, the mean score of the WFNS grading scale was 3.5, and the mean Fisher grade was 3.6. Patients with aSAH were treated with endovascular coiling (81%) or surgical clipping (19%). The two patients without aSAH were treated conservatively.

The baseline samples were obtained on day 1 from one patient, on day 2 from four patients and on day 3 from 13 patients. Five patients developed cerebral vasospasm during the first week. None developed cerebral vasospasm later than on day 8. Three patients died, of which one developed cerebral vasospasm during the trial period. Two patients developed bacterial ventriculitis during the trial period.

When comparing patients who developed cerebral vasospasm with those who did not, there were no significant differences in GCS score at admission, Fisher grade or WFNS grade. Mean age was slightly, but not significantly, higher in patients that developed cerebral vasospasm (Table 1). The mean serum S100B level in patients who developed cerebral vasospasm compared to those who did not, was lower within the first 5 days after ictus. Following day 5, serum S100B levels in the group with cerebral vasospasm, was increased and exceeded the mean level of serum S100B in patients who did not developed vasospasm, though not statistically significant (Table 2). The mean CSF S100B levels in patients who developed cerebral vasospasm were lower compared to patients without vasospasm, but not statistically significant. The peak and mean body temperatures were generally high, but did not differ between those who developed vasospasm and those who did not.

**Table 1:** **Characteristics of patients with cerebral vasospasm compared to patients without**.

	Cerebral vasospasm *n* = 5	No cerebral vasospasm *n* = 13
Gender (M:F)	2:3	5:8
Mean age (years)	67	58
Mean GCS at admission	8	9
Mean WFNS grading score at admission	3.6	3.5
Mean Fisher grading score	3.6	3.5
Mean body temperature (°C)	38.0	38.0
Intracranial infection	0	2
Death	1	2
Sample collection (*n*)
Baseline
Day 1	1	0
Day 2	1	3
Day 3	3	10
Days 5–8	5	13

*GCS, glasgow coma scale; WFNS, World federation of neurological societies grading scale*.

**Table 2:** Mean serum and CSF S100B levels in patients with cerebral vasospasm compared to patients without*.

Samples	Mean serum S100B (μg/L)		Mean CSF S100B (μg/L)	
	Cerebral vasospasm *N* = 5	No cerebral vasospasm *N* = 13	Cerebral vasospasm *N* = 5	No cerebral vasospasm *N* = 13
Baseline	0.10	0.19	23.5	29.6
Day 5	0.15	0.37	13.8	21.8
Day 6	0.21	0.15	10.1	16.3
Day 7	0.27	0.11	12.7	15.7
Day 8	0.24	0.25	12.0	12.3

*There are no statistical differences between the two groups (vasospasm vs. no vasospasm).

**Figure 1:**
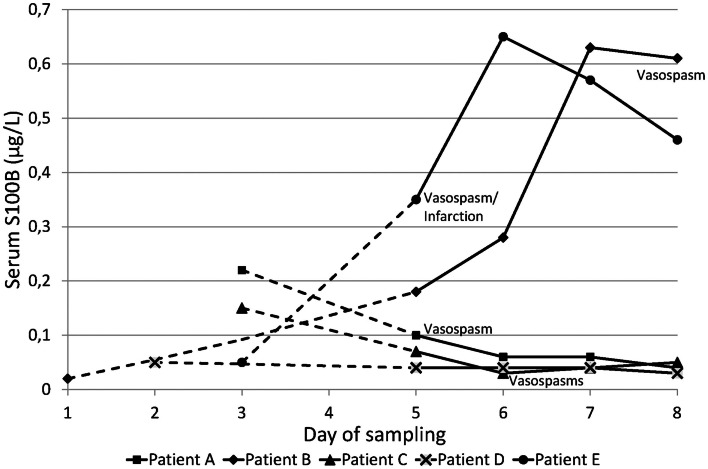
**Serum S100B levels for the five patients with cerebral vasospasm**. Day 1 = day of ictus. Baseline sampling was made between day 1 and 3. The dotted lines are extrapolations from baseline sample to the second sample drawn on day 5. The day on which cerebral vasospasm was confirmed is indicated for each patient.

Among the five patients who developed cerebral vasospasm, baseline samples of serum and CSF S100B were drawn on day 1 in one patient, at day 2 in one patient, and at day 3 in three. Two patients had increasing levels of serum S100B compared to the other three, which had an overall decreasing tendency and normal levels of serum S100B when vasospasm was diagnosed. Only one patient reached a peak level of serum S100B the day before vasospasm was confirmed (Figure 1, patient B), while the other patient reached peak S100B 1 day after angiographic confirmation of cerebral vasospasm and ongoing cerebral infarction (Figure 1, patient E). Both patients had a WFNS score of 5 and a GCS of 3 at admission, and both developed pneumonia during the trial period. The other three patients with cerebral vasospasm had slightly lower WFNS and slightly higher GCS scores at admission, and none had pneumonia or other infections diagnosed during the trial period.

Among patients who did not developed cerebral vasospasm, the baseline samples were drawn on day 2 in three patients and on day 3 in 13 patients. Two developed bacterial ventriculitis, with a sudden 10-fold increase in serum S100B levels. One reached peak level of serum S100B the day before developing ventriculitis, while peak levels for the other patient occurred on the same day as the infection was diagnosed. No such relation was observed for CSF S100B.

## Discussion

We studied a total of 18 SAH patients by measuring S100B in serum and CSF within the first 8 days following ictus. Five patients developed cerebral vasospasm, detected by neurological deterioration and confirmed with CTA. No significant differences for S100B levels were observed when comparing patients who developed cerebral vasospasm to patients who did not, and S100B failed predict the development of cerebral vasospasm.

Only two patients with cerebral vasospasm developed increased levels of serum S100B during the trial period. Both patients were in worse neurological condition (GCS 3, WFNS 5 at admission) compared to the other patients who developed cerebral vasospasm (Stranjalis et al., 2007). In addition, these two patients developed pneumonia during the trial period. Two patients in the group without cerebral vasospasm developed ventriculitis. High levels of serum S100B were found on the day before or on the same day as the clinical diagnosis of bacterial ventriculitis was set. The results indicate an association between increased serum S100B and intracerebral infections as shown in a previous study (Unden et al., 2004).

Measurements of S100B in our patients were done once daily with baseline sample obtained within the first 3 days of ictus and the rest of the four samples on days 5–8 following ictus. The baseline samples were obtained within the first 3 days of ictus, as cerebral vasospasm usually do not occur before day 4 following ictus. As the highest risk for developing vasospasm is between 6 and 8 days following the subarachnoid bleeding (Weir et al., 1978), the subsequent samples were drawn on day 5–8. None of the patients developed either clinical or angiographic vasospasm after day 8.

In two patients the bleeding source was not an aneurysm, but a small deep AVM in one patient, and a vertebral artery dissection in the other. The risk of developing cerebral vasospasm increases with increasing volume of subarachnoid blood visualized by CT (Fisher et al., 1980; Adams et al., 1987). Both of these patients had high amount of subarachnoid blood visualized on CT (Fisher grade 3 and 4), and thus high risk of developing cerebral vasospasm.

Currently, neurosurgical centers use daily assessment of neurological status, TCD sonography and CTA to determine the development of cerebral vasospasm. Most patients suffering from severe SAH, have already, prior to transport to the NICU, been sedated and intubated, making the assessment of the neurological status in these patients challenging. In our study, clinical suspicion of vasospasm in addition with CTA, rather than TCD, was used to diagnose cerebral vasospasm. CTA has been found to correlate well (overall agreement of 95%) with DSA (Yoon et al., 2006), which has been considered as the gold standard in detecting cerebral vasospasm. Furthermore, Kunze et al. (2012) have shown that the accuracy of neurological examination and CTP is higher in detecting cerebral vasospasm than TCD, and in a systematic review by Lysakowski and colleagues, they concluded that although TCD has a high specificity (0.99) and positive predictive value (0.97) for detecting vasospasm, this accounts only for the middle cerebral artery. For all the other arteries, there is no evidence for the usefulness of TCD as a diagnostic tool for vasospasm (Lysakowski et al., 2001).

Our study also show a tendency toward lower serum S100B levels during the first 5 days following SAH in patients who developed cerebral vasospasm compared to those who did not. These results were not statistically significant, thus not supporting useful value of serum S100B in predicting vasospasm. Similar results have been found by Oertel et al. (2006) who showed lower levels of serum S100B within the first 3 days after SAH in patients who developed cerebral vasospasm compared to those who did not. Cerebral vasospasm was determined by neurological deterioration and increasing flow velocity on TCD.

Identifying a specific biomarker for prediction of cerebral vasospasm in this group of patients is of high value, since earlier detection and hence earlier treatment of vasospasm could lower the morbidity and mortality in this group of patients. The statistical analysis of our end results are limited by the small number of cases enrolled in the study. We can, however, conclude that, although S100B is a promising prognostic biomarker of secondary brain damage and outcome in patients with SAH (Stranjalis et al., 2007; Sanchez-Pena et al., 2008), the potential of S100B as a predictor of cerebral vasospasm is very limited. This is in accordance with two previous studies (Moritz et al., 2010; Jung et al., 2013). The need of a marker in predicting cerebral vasospasm still remains, and other biomarkers such as myeloperoxidase (Lim et al., 2012), amino acids in addition to microdialysis (Jung et al., 2013), endothelin-1, interleukin-6, and indicators of thrombin activity might be of greater utility (Lad et al., 2012).

## Conflict of Interest Statement

The authors declare that the research was conducted in the absence of any commercial or financial relationships that could be construed as a potential conflict of interest.
